# Electronic Waste Low-Temperature Processing: An Alternative Thermochemical Pretreatment to Improve Component Separation

**DOI:** 10.3390/ma14206228

**Published:** 2021-10-19

**Authors:** Juliana S. S. Oliveira, Ronald R. Hacha, Felipe S. d’Almeida, Caroline A. Almeida, Francisco J. Moura, Eduardo A. Brocchi, Rodrigo F. M. Souza

**Affiliations:** Department of Chemical and Materials Engineering, Pontifical Catholic University of Rio de Janeiro, Rio de Janeiro 22451-900, RJ, Brazil; juliana.sette@yahoo.com.br (J.S.S.O.); rojas@esp.puc-rio.br (R.R.H.); felipe_dalmeida@hotmail.com (F.S.d.); caroline.azvdo@gmail.com (C.A.A.); moura@puc-rio.br (F.J.M.); ebrocchi@puc-rio.br (E.A.B.)

**Keywords:** low-temperature processing, WEEE (waste of electric and electronic equipment) recycling, materials separation, process characterization, SEM/EDS

## Abstract

The production of electronic waste due to technological development, economic growth and increasing population has been rising fast, pushing for solutions before the environmental pressure achieves unprecedented levels. Recently, it was observed that many extractive metallurgy alternatives had been considered to recover value from this type of waste. Regarding pyrometallurgy, little is known about the low-temperature processing applied before fragmentation and subsequent component separation. Therefore, the present manuscript studies such alternative based on scanning electron microscopy characterization. The sample used in the study was supplied by a local recycling center in Rio de Janeiro, Brazil. The mass loss was constant at around 30% for temperatures higher than 300 °C. Based on this fact, the waste material was then submitted to low-temperature processing at 350 °C followed by attrition disassembling, size classification, and magnetic concentration steps. In the end, this first report of the project shows that 15% of the sample was recovered with metallic components with high economic value, such as Cu, Ni, and Au, indicating that such methods could be an interesting alternative to be explored in the future for the development of alternative electronic waste extraction routes.

## 1. Introduction and Literature Review

The production of urban waste is becoming a subject of environmental pressure in modern society with impacts of sanitary, social, and financial importance [1,2,3]. The observed economic growth and population increase of recent decades have pushed for the manufacturing of more products and devices, intensifying issues associated with environmental pollution and depletion of natural resources, and are now driving initiatives related to the treatment of this class of wastes [4,5,6,7] In this context, the waste of electric and electronic equipment (WEEE) is considered a critical byproduct of urban lifestyles [8,9,10]. WEEE is an unconventional waste, typically with high metal content that is challenging to recycle based on traditional metallurgical processes [9].

WEEE has a wide variety of different components and devices, the most common being copper wire, batteries, structural components, LCD screens and printed circuit boards (PCBs). The mass percentage of the components (e.g., metal, polymers, and ceramics) varies a lot with the type of equipment and brand [11,12]. From a resource perspective, this type of waste has higher concentrations of metals than those found in the typical run-of-mines (ROM) [13], making WEEE recycling a possible secondary source of metals [14,15]. From an economical perspective, it was estimated that in 2017, WEEE accumulated a worldwide value of EUR 55 billion in raw material [16,17]. Thus, it is necessary to develop technological routes and managements policies to consolidate the recycling of WEEE to recover valuable materials [18]. High levels of metal recovery from WEEE have been reported and the costs associated with it are becoming more competitive but are still higher than those associated with mining operations [5,19]. In terms of economic potential in Brazil, a previous work from our research group estimated, based on a population survey and mass balance, that the stockpile value of devices in hibernation could be as high as USD 797 million [20].

In parallel to this context, PCBs are present in every electronic device, representing about 3 to 7% of the equipment’s mass [21,22,23,24]. Computer-based PCBs are composed, essentially, of an epoxy resin or fiberglass coated with a thin layer of copper and are classified according to the composition of the insulator used. Fire-resistant material made of fiberglass and epoxy is the most used today [25]. In addition to Cu, PCBs contain a wide variety of metals, for example, Au, Pd, Ag and Ni [26]. However, hazardous metals such as Cd, Pb and Be may also be present [27]. Therefore, many kinds of research have been carried out to the recover precious metals and to the remove harmful elements/compounds [28,29,30,31]. In addition to that, there are also some environmental sustainability issues associated with the already-established routes which need to be faced in the coming years [32].

The combination of chemical and physical methods is a commonly used route in WEEE recycling. However, due to cost associated with chemical inputs and easy operation, most of the PCBs are processed using incineration and acid leaching, producing a substantial amount of hazardous emissions [33,34,35]. Moreover, poor WEEE disposal and processing could be related to the production of a variety of dangerous compounds, such as dioxins, that could be responsible for serious health issues [16,36,37]. Additionally, there are also concerns associated with persistent pollutants from the polymeric contents of the WEEE [10], such as polycyclic aromatic hydrocarbons [38], polychlorinated biphenyls [39,40], polychlorinated dibenzo-p-dioxins and dibenzofurans [40,41], and brominated and organophosphate compounds [39,42]. In this perspective, low-temperature processing-related processes could be regarded as appealing options to deal with these risks [23,32,43].

Typically, regarding thermal processing, most WEEE recycling proposals start with physical beneficiation, essentially disassembling and grinding the PCBs samples [23,44]. This mimics a typical extractive metallurgy approach, with the same energy-intensive requirements to reduce the particle size. Another disadvantage of this type of physical processing is the fact that it is not possible to obtain a pure material in its present form, and the consequent production of fine comminution powder [45]. These two conditions present a challenge to the cost. According to Quan et al. (2010) [27], excessive fragmentation limits the recovery of fiberglass and can significantly increase metal losses while imposing a high energy consumption for the operation of the fragmentation equipment due to the high hardness of the PCBs. Nevertheless, the high-temperature processing routes generate products that can be recovered and reused [23,27,46,47]. The review by Ambaye et al. (2020) [48] showed that WEEE recycling by means of pyrometallurgy is an energy-intensive alternative, majorly focused on copper recovery. Moreover, it seems that little is known on the effect of pyrometallurgical processes being applied before the initial physical processing, particularly regarding the investigation of low-temperature processing effects on PCB constituent separation. For instance, Ma et al. (2018) [49] assessed this type of processing from a heat transfer perspective while Guo et al. (2014) [50] dealt with calorific capacities of a PCB sample. In praxis, the typical pyrometallurgical processes are furnace smelting and alkali fusion, according to Chauhan et al. (2018) [35] and Ding et al. (2019) [51]. Additionally, thermal processing of WEEE is regarded as a promising alternative for recycling the non-metallic fractions [52].

Therefore, the present work has the motivation of providing a first look into such alternatives, to offer conditions for easy-to-implement physical disassembling without major particle size reduction. The technical support of such proposition is related to previous observations in which the thermal processing was investigated to produce a solid, free of the volatile organic fraction, to hydrometallurgical leaching without excessive fragmentation [53]. The possibility of a processing alternative without the prior comminution, bypassing the initial physical beneficiation step, was also reported for PCBs with the chemical characterization of the oil-based resin [27].

The present study has the interest of exploring some of the material’s behavior in a pyrometallurgical process through electron microscopy characterization of the resulting materials, as it seems that the most relevant effort in material characterization has been reported for hydrometallurgical or for pure mineral processing approaches [54,55,56].

The technological context of this proposition is associated with the present Brazilian context, in which some important initiatives towards WEEE collection, disassembling and parts recycling can be observed but with little advance in material recovery through chemical processing [57,58,59,60]. According to Nithya et al. [61] Brazil is among the top five 2019 WEEE producers after China, USA, India and Japan, producing more than 2 million tons per year [62]. Moreover, it is also recognized as a trans-boundary destination of electronic wastes with lack of proper infrastructure related to waste management [63]. Dias et al. [64] present an alarming scenario in which the Brazilian recycling system operates towards valuable constituent concentration and undertakes shipping abroad to further processing of materials.

Under this perspective, the present manuscript’s purpose is related to the investigation of the PCBs material’s behavior in a low temperature pyrometallurgical processing operation, prior to physical fragmentation based on scanning electron microscopy (SEM/EDS) characterization. The study also covers a thermogravimetric analysis (TGA) to identify the lower temperature in which the process could be carried out to provide material disassembling without major fragmentation.

## 2. Materials and Methods

Samples of connectors from hard-disk drives (HD) and random-access memories (RAM) were received from a local recycling center in Rio de Janeiro, Brazil, that operates with the collection, disassembly, and parts separation for reuse. The samples were collected from the supplier stockpile of non-recyclable parts and locally processed to concentrate valuable metallic content.

**Figure 1:**
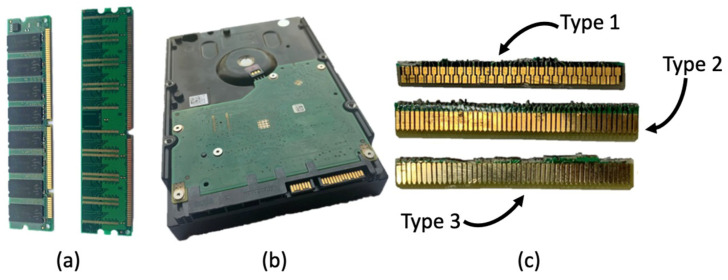
Macroscopic features of the received WEEE sample: (**a**) RAM; (**b**) HD; (**c**) Varieties of contacts from RAM and HD as received.

After receiving the sample, a visual classification was carried out and three patterns of connectors that were categorized as Type 1, Type 2 and Type 3 varieties. In terms of the visual distinctions of each variety, it was observed that Type 1 and 2 were presented with a more distinguished goldish yellow with differences in the morphology of the metallic parts. On the other hand, Type 3 presents a pale gold color with similarities in contact shape with Type 2. Figure 1 shows the macroscopic features of the received material. The WEEE samples, as well as all the solid materials produced in this study, were characterized using scanning electron microscopy coupled with energy-dispersive X-ray spectroscopy (SEM/EDS), using a Hitachi TM3000 microscope (Hitachi, Tokyo, Japan) connected with an Oxford Swift ED3000 microanalysis system. The detection mode for SEM is Backscattered Electrons. The thermogravimetric analysis (TGA) was carried out on NETZSCH STA 449 F3 Jupiter equipment (NETZSCH, Selb, Germany) using a 10 K·min^−1^ heating from room temperature until 1000 °C. The WEEE samples had their thermal behavior evaluated under a chemically inactive as well as oxidizing atmosphere. The former was conducted with high-purity nitrogen (99.98%) while the latter was taken into effect with a synthetic air mixture composed of 80% N_2_ and 20% O_2_. Both gas mixtures were manufactured by Linde company (Dublin, Ireland). The applied flowrate entering the reaction chamber was fixed at 20 L·min^−1^.

**Figure 2:**
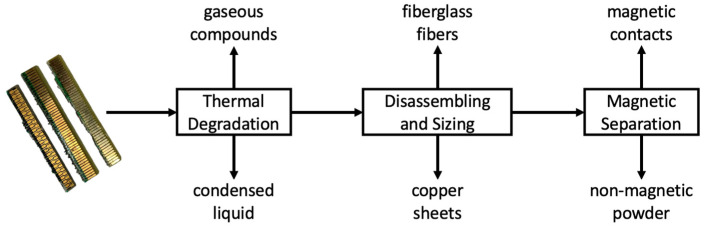
The proposed chemical processing route for the received WEEE samples.

After the WEEE microscopical and thermal characterizations, samples were submitted to the chemical process of low-temperature processing, physical disassembling with glass bodies, size classification and magnetic separation. Figure 2 presents a schematic representation of the proposed route to process WEEE samples and recover valuable metals. The sequence of unit operation was defined to avoid major shredding and fine powder formation, as the pyrometallurgical process could provide the volatilization of chemicals responsible for the structural integrity of the PCBs.

Samples of WEEE were submitted to isothermal pyrometallurgical processing in a tubular furnace. The experiments were conducted in compressed air (incineration) and ultrapure argon (99.998%) atmosphere (inert processing). The latter was also supplied by Linde. It was defined that the WEEE samples would be accommodated at room temperature inside the furnace and then heated until the desired temperature was reached. The reaction time for processing was fixed at 60 min for the defined process temperature. After thermal degradation, the solid products were cooled down until 80 °C and then removed from the furnace.

The disassembling operations were conducted with a hand-operated mill using glass pebbles as friction bodies. This option was taken on purpose as it would exemplify how easily the physical detachment of constituents occurs after high-temperature processing without deleterious effects on components liberation. The produced particulate system was then collected and classified by size, using sieves with openings of 4.75, 2.80, 1.4, 0.71, 0.50, 0.21 and 0.18 mm. Finally, the small-sized fraction was exposed to a magnetic field from a hand-magnet, again because of the simplicity of the operation. The recovered materials were then characterized by SEM/EDS to assess the performance of the proposed route as an alternative to separating some of the constituents.

## 3. Results and Discussion

### 3.1. Characterization of WEEE Samples

**Figure 3:**
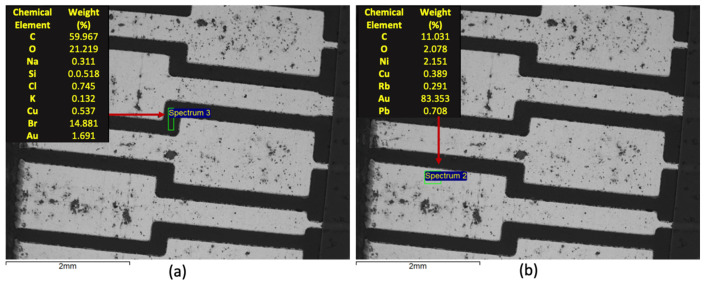
SEM/EDS characterization of Type 1 WEEE sample: (**a**) microanalysis of the dark area; (**b**) microanalysis of the bright area.

Samples of the three varieties of WEEE were submitted to SEM/EDS to identify the chemical distribution in the PCB through a semiquantitative approach. The detection mode for this study is related to Backscattered Electrons. In this context, Figure 3 presents such results for a sample of the Type 1 variety. It can be observed that as expected the metallic area of the PCB features a bright shade of grey, associated with a higher average atomic number, while the dark area is associated with the polymeric material. Some clouds of dark structures could also be observed over the bright area, possibly associated with oxidation of the metallic parts, possibly associated with nickel and copper. It is noteworthy to mention that the image and the respective EDS results are mainly associated with the surface area of the sample. In the dark area, a major presence of carbon, oxygen, and bromine can be observed, while in the bright area, gold stands as the major constituent, at least at the sample surface. These are interesting and expected findings as brominated compounds are used in this context as flame retardants, while gold layers on the surface of the metallic contacts are responsible for increasing efficient current transmission.

**Figure 4:**
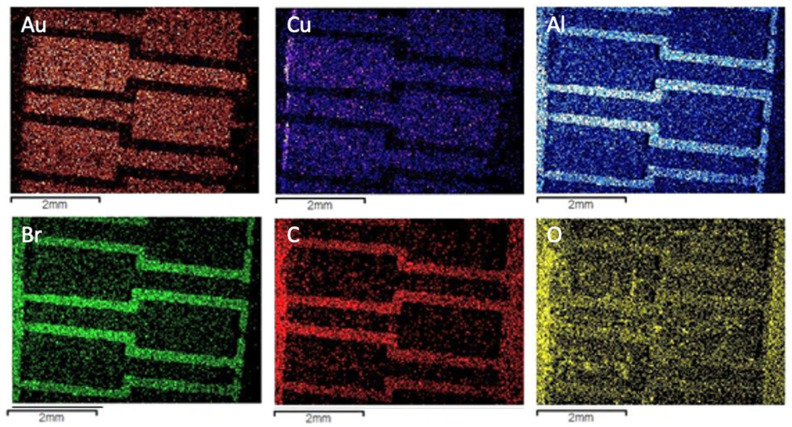
Chemical mapping of the most relevant elements detected in the microanalysis of the dark and bright areas of the Type 1 WEEE sample.

However, the amounts of other valuable metals, such as copper (in the inner contact) and aluminum (in the fiberglass) could only be qualitatively assessed by utilizing a chemical mapping that analyses the composition distribution deeper in the sample. Such analysis is presented in Figure 4.

**Figure 5:**
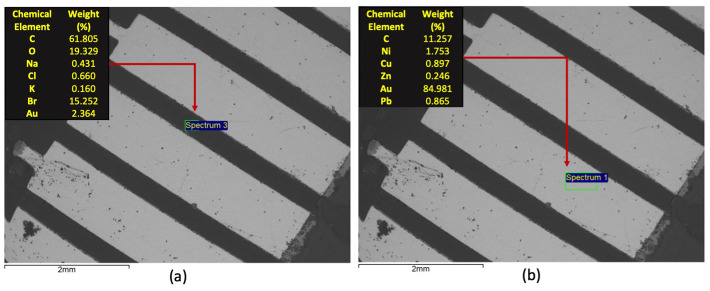
SEM/EDS characterization of Type 2 WEEE sample: (**a**) microanalysis of the dark area; (**b**) microanalysis of the bright area.

**Figure 6:**
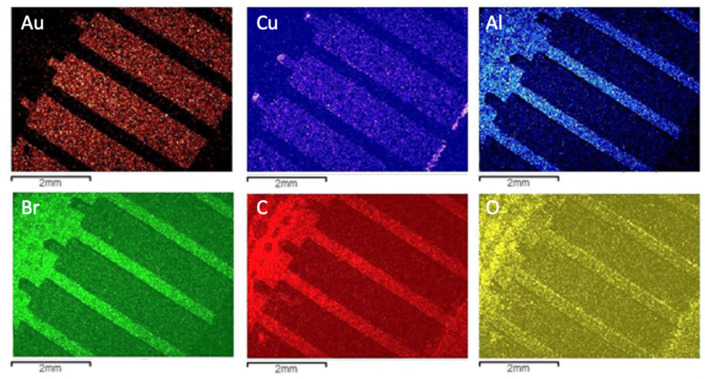
Chemical mapping of the most relevant elements detected in the microanalysis of the dark and bright areas of the Type 2 WEEE sample.

Figure 5 presents the SEM/EDS analysis while Figure 6 shows the qualitative chemical mapping of the Type 2 variety of WEEE in the received sample. It can be observed that Type 2 follows Type 1 in terms of the overall chemical composition of the bright (metallic) and darker (polymeric) areas, with a cleaner surface regarding the presence of clouds potentially associated with metal oxidation. Comparing the two varieties, at a semiquantitative level, the composition in both areas in each sample is relatable.

**Figure 7:**
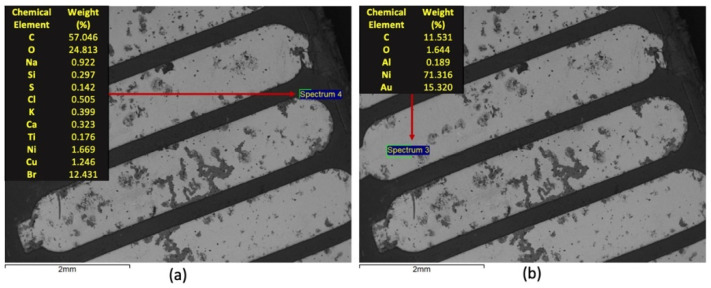
SEM/EDS characterization of Type 3 WEEE sample: (**a**) microanalysis of the dark area; (**b**) microanalysis of the bright area.

On the other hand, the Type 3 variety, with a paler metallic hue, does not follow the other two in terms of composition and surface integrity, as shown in Figure 7. It was verified that the samples of this variety present a similar overall composition for the dark (polymeric) area as the one observed in the previous cases, but with a larger number of elements in the lower range of relevance. For the bright (metallic) area, the distinction is clear, with nickel as the major metallic constituent and with gold still present at an important level. The dark clouds were most present in this variety, which could be related to lower levels of Au in the surface.

**Figure 8:**
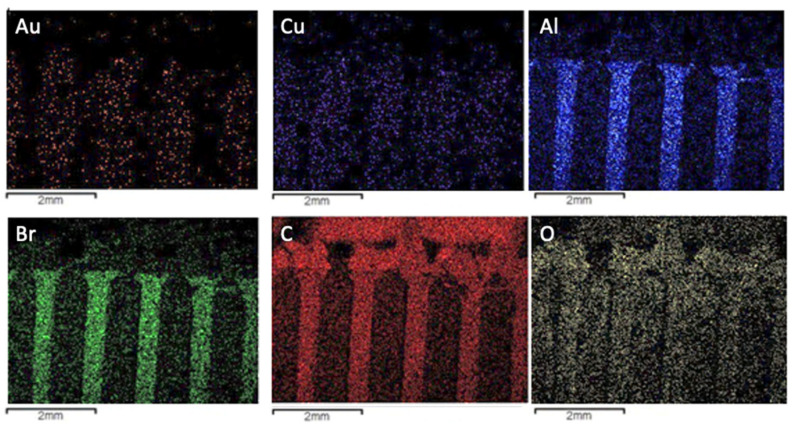
Chemical mapping of the most relevant elements detected in the microanalysis of the dark and bright areas of the Type 3 WEEE sample.

Following the same approach, Figure 8 displays the chemical mapping of the Type 3 variety. The major qualitative difference between this variety and the others is related to the lower presence of gold and the higher distribution of oxygen, which corroborates the behavior associated with the more distinguished dark clouds observed in Figure 7.

**Figure 9:**
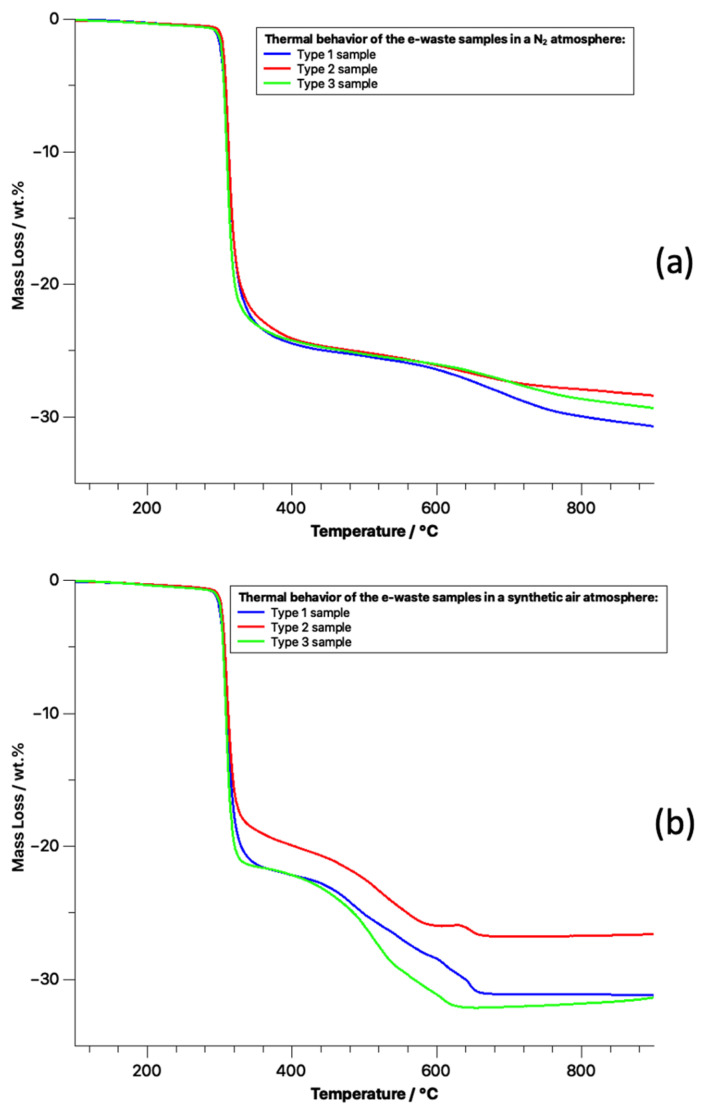
Thermal behavior characterization of WEEE sample: (**a**) in an inert atmosphere; (**b**) in an oxidizing atmosphere.

In parallel to the SEM/EDS characterization, samples of each of one of the three varieties of WEEE were also submitted to non-isothermal TGA in oxidizing and chemical inert conditions, as presented in Figure 9. In praxis, the same thermal behavior as a function of temperature regardless of PCB type and reaction atmosphere was verified. The total weight variation indicates a mass loss of 30%. Regarding the chemical environment, at N_2_ atmosphere, carbon and hydrogen were volatilized as organic compounds, possibly carrying flame-retardant components, whereas in the oxidative experiment these elements were possibly being transported to the gas phase as oxidized compounds such as water, monoxide, and carbon dioxide [65]. The observed degradation temperature is in accordance with the presence of thermoplastic materials in the sample, at about 250 °C [27].

Both materials’ characterization results indicate similarities between the three varieties, particularly regarding the polymeric fraction, and therefore to establish the chemical process more simply, the thermal processing of the received sample was considered without the variety distinction, to remove at least a fraction of the organic phase and to liberate constituents. Consequently, the thermochemical processing of the WEEE samples was carried-out for the material in the same condition as it was received from the local recycling center, without any classification, in a tubular furnace above 300 °C.

### 3.2. Thermal Processing of WEEE Samples

Table 1 shows the experimental results associated with the pyrometallurgical processing of 2.5 g of PCB connector at 300 and 400 °C, using compressed air (incineration) and argon (inert processing). As expected, the weight loss was again around 30%. However, at the inert gas atmosphere, the formation of some droplets of a black liquid at the far end of the tubular furnace were observed. This was interesting, and an indicative of the volatilization and condensation of the organic fraction, as reported previously by other authors [27,53].

**Figure 10:**
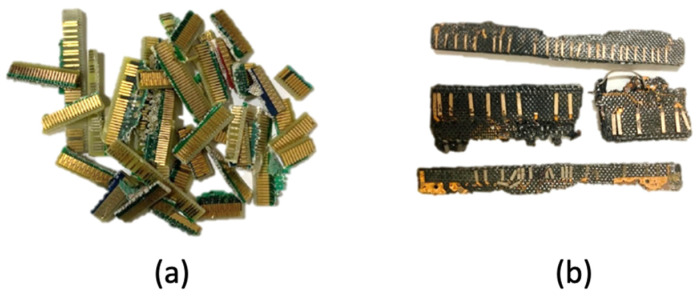
Macroscopic aspects of the WEEE samples: (**a**) before thermal processing; (**b**) after thermal processing.

To produce more liquid and to generate more solid material, another thermal degradation experiment at 350 °C was carried out in argon using 54.5 g of the WEEE sample. The reaction time of 60 min was also applied to this test. A weight loss of 30.1% was observed, resulting in a solid weight of 38.1 g. In this context, Figure 10 presents the macroscopic aspect variation before and after thermal degradation. It is noteworthy that some substance has been removed from PCBs, exposing the metallic compounds, some copper sheets as well as the inner glass fibers, now covered with some dark material, possibly carbon black.

The disassembling and sizing unit operation was applied to the solid product, and, in this context, Table 2 presents the size classification of the obtained material after these operations.

**Figure 11:**
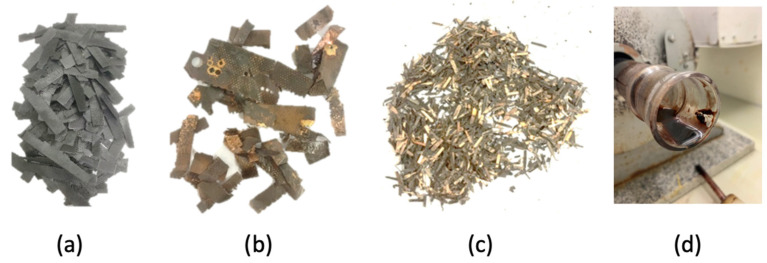
Macroscopic aspects of some relevant materials recovered in the process: (**a**) fiberglass; (**b**) copper sheets; (**c**) magnetic contacts; (**d**) condensed liquid.

It can be observed that most of the size classification is associated with large particulate material. A total of 80.71% is associated with glass fibers (17.4 g), copper sheets (1.3 g) and non-liberated material (12.0 g). The fact that 31.58% of the sample is associated with non-liberated material indicates that the disassembling unit operation could be the subject of future developments, to optimize larger recovery of metals. Moreover, the non-liberated material could also be submitted to hydrometallurgical processes as most of the organic phase has been removed from it, in a roast–leach type of route. Material below 2.80 mm was submitted to magnetic separation. It was observed that 4.7 g was susceptible to the effects of the magnetic field and recovered easily. Therefore, it can be said that 12.4% of the solid product is composed of magnetic-metal-containing materials. Figure 11 illustrates the macroscopic aspect of the most relevant materials recovered.

### 3.3. Characterization of the Thermal Processing Products

**Figure 12:**
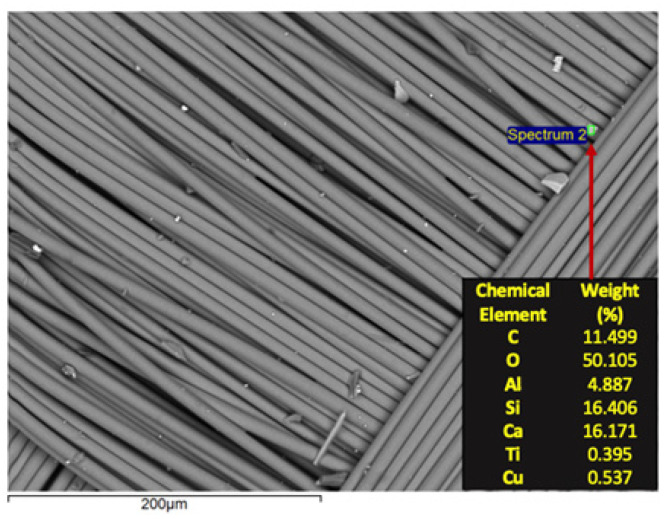
SEM/EDS characterization of the fiberglass recovered after the size separation.

The recovered glass fiber was characterized utilizing SEM/EDS and its results are presented in Figure 12. It is possible to note that Si and Ca are the major metals in the fiberglass while oxygen is the major overall component. The preeminent levels of C also followed the carbon-black-deposition expectations. Some minor contents of Al, Ti and Cu were also detected. Some minor, bright, particulate material within the knitting pattern can be observed. Since such elements are heavier than C, and combined with the Backscattered Electrons detection mode, it is possible to affirm that some metallic powder is also being transported with the fiberglass.

**Figure 13:**
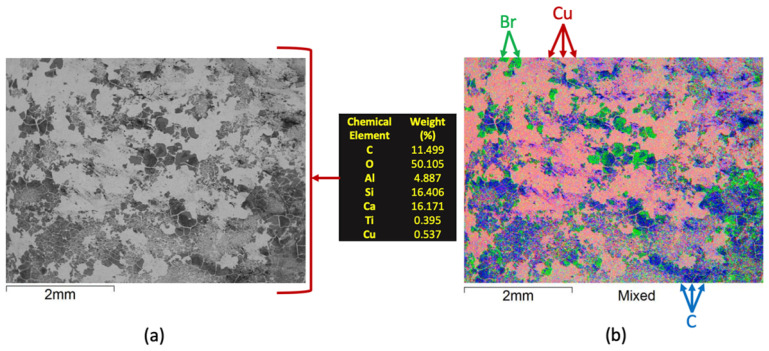
SEM/EDS characterization of the copper sheets recovered after the size separation: (**a**) Backscattered Electrons Image with microanalysis; (**b**) chemical mapping.

Figure 13 is associated with the copper sheets recovered in this study, presenting the SEM/EDS analysis as well as the chemical mapping for the same area. The morphological aspect of the image indicates some deposition of organic matter (dark scales) over the copper sheet (bright area). This is indicative that some fraction of the polymeric material is not being transferred to the gas phase in the thermal degradation. The qualitative chemical mapping clarifies that suggestion, as copper is the major component of this material while the scales have carbon and bromine in their composition. This context indicates that temperature and reaction time could also be optimized to separate copper from non-metals and flame-retardant elements.

**Figure 14:**
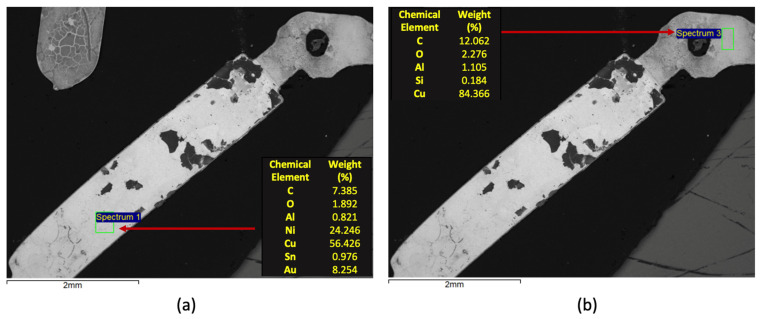
SEM/EDS characterization of the metallic contacts recovered after the size separation and magnetic separation: (**a**) microanalysis of the surface layer; (**b**) microanalysis of the inner contact.

Figure 14 presents the SEM/EDS analysis of the recovered magnetic connector. It was verified, through the EDS microanalysis, that the Cu, Ni and Au are being recovered in this material (Figure 14a). Without size reduction to powder, some non-magnetic metals remain linked to nickel providing its concentration being subjected to a magnetic field. These elements are the main constituents of the bright area of the connector that covers the inner layer of copper (Figure 14b). The presence of carbon is also perceptible, both in the EDS microanalysis as well as through the dark matter over the metallic phase.

## 4. Final Remarks

In short, the proposed route was established as an alternative for processing PCB samples and to valorize them, concentrating metals, separating these from some pf the ceramic content and organic substances. The process produced a black liquid which condensates from the gas phase, therefore diminishing atmospheric emissions and possibly providing a source of materials in some technological applications. 

The SEM/EDS analyses showed that the support for the alloy connectors was composed of carbon and oxygen, which indicates, as expected, a typical polymeric material. The presence of bromine, related to flame-retardant additives, was also observed, as were small fractions of metals. The chemical composition for this part of the PCBs samples does not change between varieties. Analyses also showed that the metal connectors have some compositional distinctions between the three types. It was observed that all three samples contain gold and nickel in the surface. The main difference is the relative amounts, with Au as the main metallic constituent for Types 1 and 2 while it is Ni for Type 3. The chemical mapping indicated a clear presence of copper in the inner layers of the connectors. The distribution of chemical elements is also clear following the SEM/EDS approach.

The TGA analyses showed a mass loss of 30%. The observed degradation temperature of about 250 °C is related to the presence of thermoplastic materials. This behavior indicates that all three samples could be processed simultaneously to volatilize the organic fraction. The tubular reactor processing also showed a mass loss of around 30% in weight. The formation of black liquid under inert processing condition was also observed. The characterization of the produced fluid will be assessed in future developments.

It is interesting to observe that combing all metallic contacts in a single fraction in the magnetic separation step is a significant contribution and a simple alternative for metals separation. It should be noted that these characterization and processing experiments could contribute to future recycling route development, lowering environmental impacts associated with this type of WEEE.

## Figures and Tables

**Table 1:** Observed WEEE samples’ mass loss after thermal processing in a tubular furnace as a function of the temperature and the atmospheric chemical composition.

Temperature above Sample in the Furnace (°C)	Mass Loss in a Compressed-Air Atmosphere (wt.%)	Mass Loss in an Ultrapure Argon Atmosphere (wt.%)
300	31.5	28.5
400	32.0	30.1

**Table 2:** Size classification of the thermal processing solid product after grinding operation using glass pebbles as friction agents for materials disassembling.

Sieve Opening (mm)	Retained Mass (g)	Retained Mass (wt.%)
4.75	30.67	80.71
2.80	0.77	2.03
1.40	0.67	1.76
0.71	4.20	11.05
0.50	0.75	1.97
0.21	0.41	1.08
0.18	0.29	0.76
Bottom	0.24	0.63
Total	38.00	100.00

## Data Availability

Data will be made available based on request to the authors.
